# Potent and selective eradication of tumor cells by an EpCAM-targeted Ras-degrading enzyme

**DOI:** 10.1016/j.omto.2023.06.002

**Published:** 2023-06-27

**Authors:** Valentina Palacio-Castañeda, Bas van de Crommert, Elke Verploegen, Mike Overeem, Jenny van Oostrum, Wouter P.R. Verdurmen

**Affiliations:** 1 Department of Medical BioSciences, Radboud University Medical Center, Geert Grooteplein 28, 6525 GA Nijmegen, the Netherlands

**Keywords:** designed ankyrin repeat protein, diphtheria toxin, epithelial cell adhesion molecule, *Pseudomonas aeruginosa* exotoxin A, Ras-Rap1-specific endopeptidase, tumor-specific targeting

## Abstract

Despite decades of efforts, an urgent need remains to develop tumor cell-selective rat sarcoma (Ras)-targeting therapies that can treat patients with Ras-driven tumors. Here we report modular engineered proteins that degrade Ras selectively in tumor cells that overexpress the tumor cell marker epithelial cell adhesion molecule (EpCAM) by fusing the Ras degrader Ras-Rap1-specific endopeptidase with the translocation domain of the *Pseudomonas aeruginosa* exotoxin A (ETA) or diphtheria toxin (DT). Redirection to EpCAM is achieved by a designed ankyrin repeat protein. In two-dimensional tumor cell cultures, complete degradation of Ras proteins after 24 h was observed with EpCAM-targeted Ras degraders fused to ETA or DT in EpCAM-overexpressing MCF7 and HCT116 cells, with median inhibition concentration values at sub-nanomolar levels. The viability of EpCAM-low non-cancerous fibroblasts remained unaffected. In a three-dimensional (3D) tumor-on-a-chip system that mimics the natural tumor microenvironment, effective Ras degradation and selective toxicity toward tumor cells, particularly with the ETA-fused constructs, was determined on-chip. To conclude, we demonstrate the potential of modular engineered proteins to kill tumor cells highly selectively by simultaneously exploiting EpCAM as a tumor-specific cell surface molecule as well as Ras as an intracellular oncotarget in a 3D system mimicking the natural tumor microenvironment.

## Graphical abstract

**Figure:**
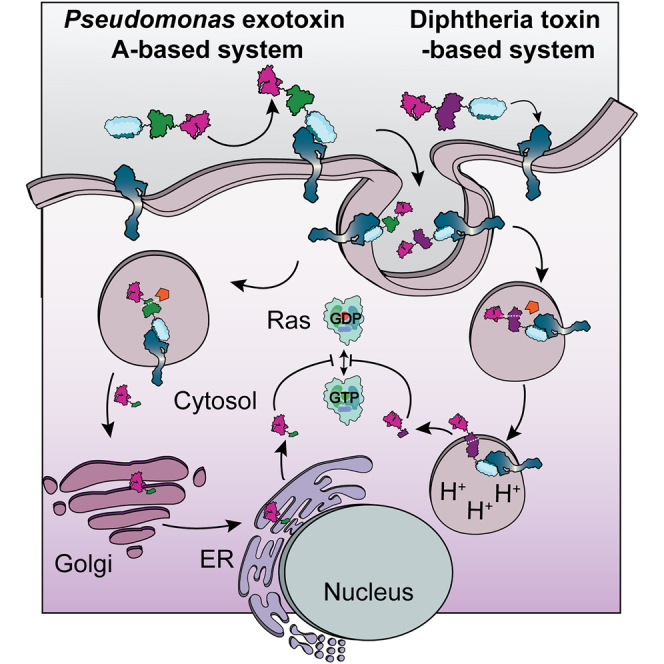

## Teaser

Verdurmen et al. show cell type-specific retargeting of the oncogene Ras, achieved by receptor targeting using a binding protein and for two translocation domains. Selective Ras-degrading activity is robustly shown both in two-dimensional and in a three-dimensional microfluidic model mimicking the tumor microenvironment.

## Introduction

Rat sarcoma (RAS) oncogenes have been extensively studied for the past three decades because of their crucial roles in malignant transformation and tumor progression. These oncogenes, encoded by three RAS genes, produce four Ras proteins: two KRas variants, NRas and HRas. Approximately 30% of all human cancers exhibit mutations in Ras oncogenes, predominantly characterized by gain-of-function mutations in codons 12, 13, and 61, resulting in continuous signaling through growth and survival-promoting pathways.^1^^,^^2^ So far, no Ras-targeting therapies have been approved besides the approval of sotorasib and adagrasib for the treatment of KRAS^G12C^-mutant non-small cell lung cancer. It is important to note that the KRAS^G12C^ mutation accounts for only 11% of all KRAS mutations across different tumors.^3^ Yet, benefitting from recent studies on the structure, signaling, and function of Ras proteins, many new therapeutic approaches addressing Ras are currently under development.^4^

Developing a tumor-selective Ras-targeted therapy is highly challenging because of the limited ability to design small molecules that specifically target mutated Ras variants, particularly because of the absence of unique, sufficiently large, and deep hydrophobic pockets.^5^^,^^6^ Additionally, increased activation of wild-type Ras is observed in many tumors, alongside the ubiquitous normal expression of Ras isoforms across all tissues, promoting tumor growth even in the absence of specific targetable mutations. Consequently, the design of a pan-Ras inhibitor that can effectively inhibit all Ras isoforms in RAS-driven tumors while avoiding a body-wide shutdown of Ras signaling and intolerable side effects is highly desirable.

A recent study introduced a chimeric toxin consisting of the Ras-Rap1-specific endopeptidase (RRSP) fused to the protein translocation domain of diphtheria toxin (DT) that irreversibly cleaves and inactivates Ras proteins at low picomolar levels in two-dimensional (2D) cell cultures.^7^ By using this chimeric toxin, which binds to the natural receptor of DT, heparin-binding epidermal growth factor-like growth factor, the authors demonstrated decreased cell viability of tumor cells in 2D cultures, three-dimensional (3D) tumor spheroids, and tumor xenografts in mice after pan-Ras degradation. However, the specificity of this approach is inherently limited by the distribution of the native DT receptor. To enhance specificity in 3D tumor microenvironments or *in vivo*, a more tunable approach could potentially be achieved by using a binding protein scaffold that can be redirected to essentially any cell surface receptor.

In this study, we aim to retarget RRSP to tumor cells using designed ankyrin repeat proteins (DARPins) as a versatile retargeting platform. DARPins are highly stable binding proteins that can be selected to bind to virtually any target and are approximately 10 times smaller than conventional antibodies.^8^ Previous studies, including our own, have demonstrated the ability of DARPins to retarget cargoes to specific cell surface receptors with a high degree of specificity, such as epithelial cell adhesion molecule (EpCAM), human epidermal growth factor receptor 2, and MET.^8^^,^^9^^,^^10^^,^^11^ In this research, we focus on targeting the representative tumor cell marker EpCAM using the extensively validated DARPin Ec1.^9^^,^^12^^,^^13^ EpCAM is frequently expressed on tumor cells of epithelial origin, while its expression and accessibility in normal epithelial tissue are limited.^14^

To deliver RRSP into the cytosol, we explore the translocation domain of both the DT toxin and *Pseudomonas aeruginosa* exotoxin A (ETA). ETA is a protein toxin consisting of three domains: a receptor-binding domain, a translocation domain and a catalytic domain. By exploiting retrograde transport, in which the translocation domain linked to the catalytic domain is transported to the endoplasmic reticulum upon binding the KDEL receptor after cleavage by a furin-like protease, ETA hijacks the host (retro)translocation machinery to deliver the catalytic domain into the cytosol.^15^ Previous studies, including our own, have successfully used the ETA translocation domain in modular proteins, replacing the receptor-binding and catalytic domains, to achieve efficient receptor-targeted delivery of various protein cargoes to the cytosol.^15^^,^^16^ In this study, we evaluate the Ras-degrading ability and tumoricidal effects of our constructs in both 2D and a 3D microfluidic tumor-on-a-chip, which provides a more accurate representation of the natural human tumor microenvironment and incorporates many of the barriers encountered by engineered proteins *in vivo*.^17^ We demonstrate a high level of specificity toward tumor cells overexpressing EpCAM and observe superior potency and specificity with the ETA translocation domain-based construct compared with the DT translocation domain-based construct previously explored for targeting RRSP to the natural DT receptor.

Altogether, our study presents a potent and tunable approach to achieve two-layer specificity in tumor targeting, exploiting EpCAM as an extracellular target and Ras as an intracellular target, resulting in exceptional specificity toward Ras-addicted tumor cells, even in the absence of activating mutations.

## Results

### Tumor-selective degradation of Ras in 2D cell cultures

**Figure 1:**
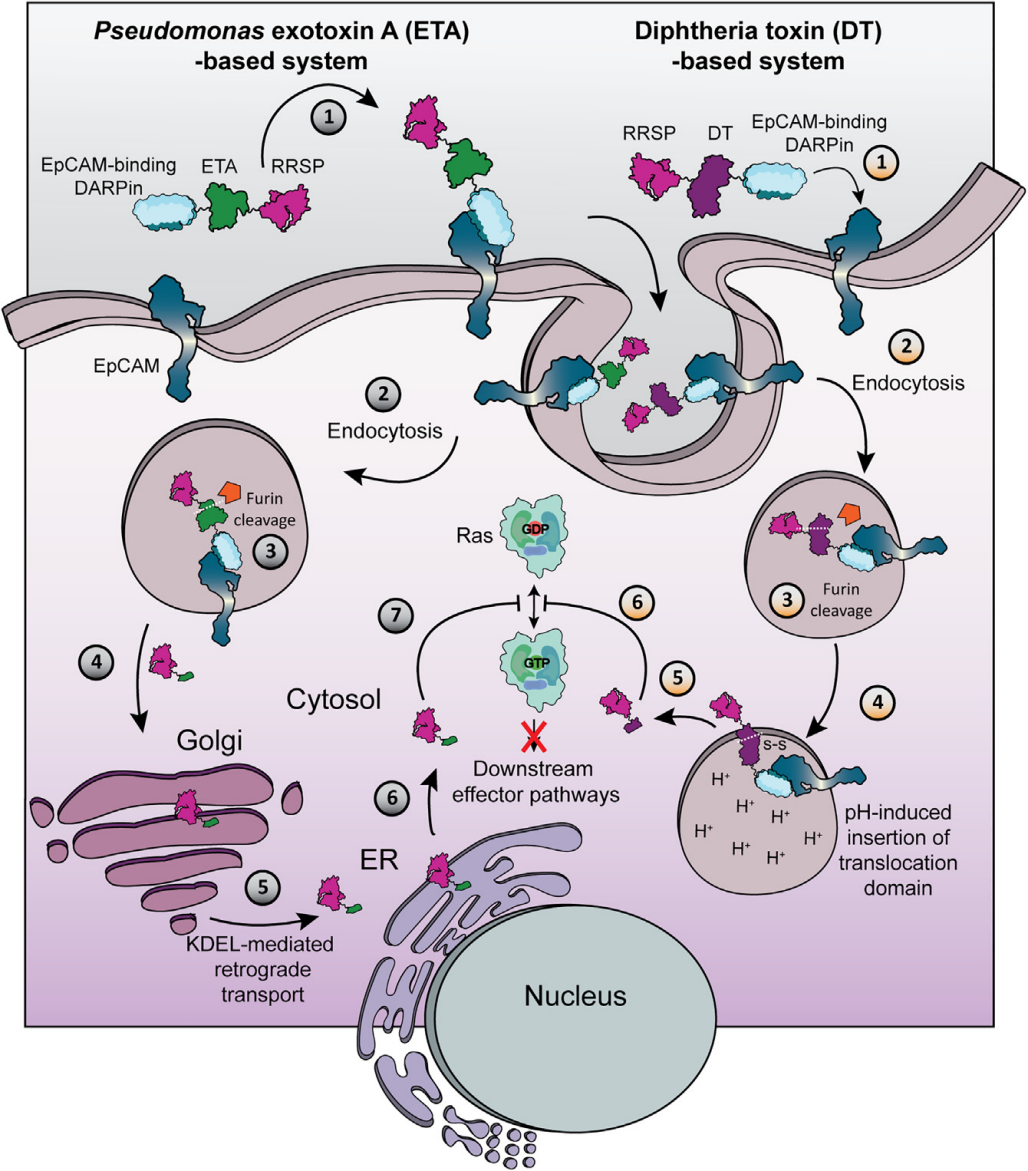
EpCAM-targeted cytosolic delivery of RRSP using the translocation mechanisms of ETA and DT For both constructs, step 1 is to bind to EpCAM which gets internalized via endocytosis in step 2. For the ETA-based system, cleavage by furin of the translocation domain occurs in the endosome which after further reduction of a disulfide bond allows for dissociation of the RRSP from the rest of the protein. In steps 4 and 5, the RRSP gets transported to the Golgi and the endoplasmic reticulum (ER) via retrograde transport mediated by the C-terminal KDEL sequence. Once in the ER, the RRSP gets transported into the cytosol in step 6. For the DT construct the translocation domain is cleaved by furin in the endosome where the low pH enables the insertion of the translocation domain in the endosomal membrane in step 4 and the reduction of the disulfide bridge allows the release of the RRSP to the cytosol in step 5. Both constructs cleave Ras proteins in step 6/7 inhibiting the ability of Ras to cycle from its inactive form (GDP bound) to its active form (GTP bound), blocking further downstream effector pathways. GTP, guanosine triphosphate; GDP, guanosine diphosphate.

To generate tumor cell-selective agents that can degrade Ras, we created modular constructs that contain Ec1 for targeting EpCAM, the Ras-degrading enzyme RRSP as active cargo, and either the translocation domain from DT (RRSP-DT-Ec1) or ETA (Ec1-ETA-RRSP). The mode of cytosolic delivery and action for the active constructs are schematically depicted in Figure 1. As negative controls, we included similar constructs that instead contained a catalytically dead RRSP, termed RRSP∗, which has a point mutation (H451A) that prevents Ras cleavage.^18^ All proteins were expressed at high levels in *Escherichia coli* and purified by immobilized metal ion affinity chromatography (IMAC) using established procedures (Figure S1A). The ability to degrade Ras proteins was confirmed *in vitro* using pure KRas. Active proteins efficiently and fully cleaved Ras (Figure S1B), which was previously linked to its inactivation.^7^^,^^18^ The catalytically dead proteins did not affect KRas.

We used MCF7 and HCT116 as targetable model cancer cell lines to investigate the tumor cell selectivity of the engineered proteins. Both cell lines express high levels of EpCAM and are Ras addicted, meaning that they rely on Ras signaling for survival. MCF7 has amplified NRAS expression and high overall Ras expression.^19^ HCT116 has a KRAS G13D mutation, which is a common gain-of-function mutation.^20^ As healthy controls, we used C5120 cells, which are normal primary human skin fibroblasts that display low EpCAM expression and no Ras alterations.

**Figure 2:**
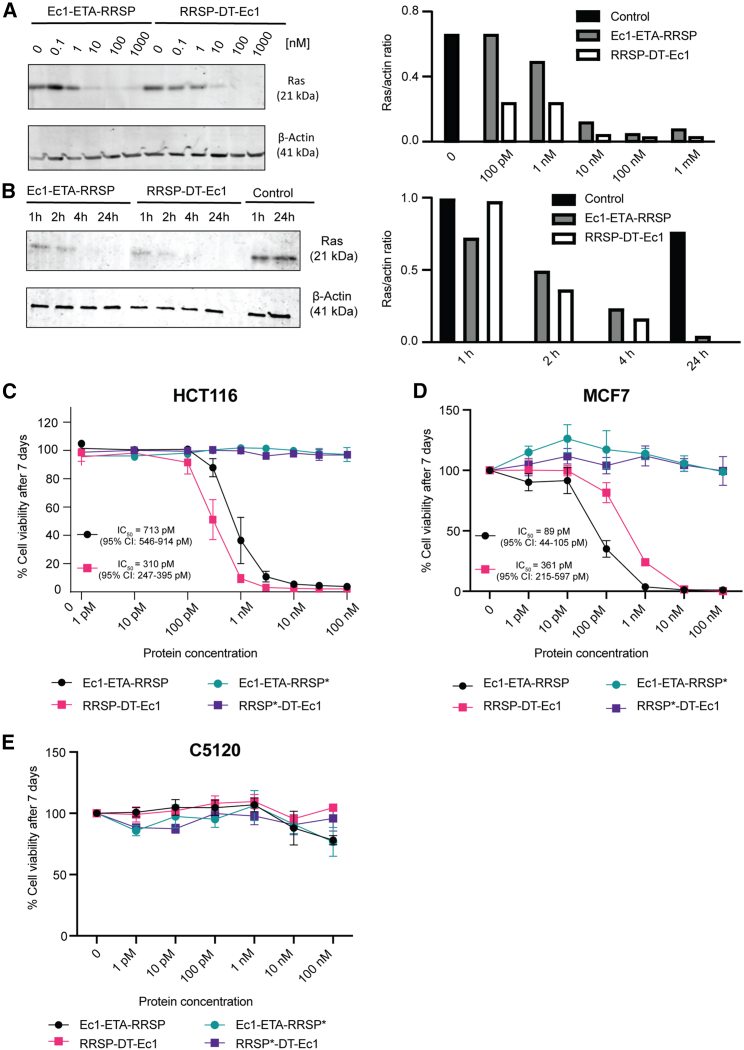
Effects of RRSP-fusion proteins on Ras levels and viability (A) Western Blot and quantification of the levels of β-actin and Ras proteins after incubation with different concentrations of Ec1-ETA-RRSP and RRSP-DT-Ec1 after 4 h of incubation in MCF7 cells (n = 3) A representative blot is shown. β-Actin (42 kDa) was used as a loading control. Ras proteins are detected at 21 kDa. (B) Western blot and quantification of Ras in MCF7 cell lysates after incubation with Ec1-ETA-RRSP and RRSP-DT-Ec1 at 100 nM or untreated control for different time points (n = 3). A representative blot is shown as in (A). (C) Cell viability of HCT116 cells after incubation with indicated concentrations of the proteins Ec1-ETA-RRSP, Ec1-ETA-RRSP∗, RRSP-DT-Ec1, and RRSP∗-DT-Ec1 (n = 3). RRSP∗ refers to a catalytically dead version of RRSP with the point mutation H451A. (D) Cell viability of MCF7 cells after incubation with different concentrations of the protein constructs (n = 3). (E) Cell viability in C5120 cells after incubation with different concentrations of the protein constructs (n = 3). Results in (C–E) are expressed as mean ± SEM.

To examine the ability of the Ec1-RRSP fusions to degrade Ras proteins in the cytosol of tumor cells, we incubated MCF7 cells with different concentrations of RRSP-DT-Ec1 and Ec1-ETA-RRSP for 4 h. Virtually complete degradation of Ras was observed at a concentration of ≥10 nM with both RRSP-DT-Ec1 and Ec1-ETA-RRSP (Figure 2A). These results demonstrate that RRSP can be effectively translocated into the cytosol when retargeted to EpCAM with the DARPin Ec1 using either the translocation domain of ETA or that of DT with apparent equal efficiencies, where it efficiently degrades Ras proteins. The ability of both constructs to degrade Ras efficiently was further validated in HCT116 cells (Figure S1C) and in EpCAM-overexpressing MDA-MB-468 and in FlpIn-293-EpCAM cells (Figure S2).^16^

The time dependency of Ras degradation was investigated in a 2D monolayer of MCF7 cells. Both Ec1-ETA-RRSP and RRSP-DT-Ec1 rapidly decreased the levels of Ras present in MCF7 cells, with lower levels detected already after 1 h, which further decreased to undetectable levels after 4 h for both constructs (Figure 2B).

We hypothesized that blockade of Ras signaling would not lead to an immediate induction of apoptosis in addicted cell lines, but rather slowly starve them of pro-survival signaling processes. Visual inspection of cells by microscopy supported the notion that therapeutic effects were most notable after approximately 7 days, which led us to evaluate the effects on viability 7 days after incubation with the engineered proteins. In MCF7 cells and HCT116 cells, the Ec1-RRSP fusion proteins decreased cell viability in a concentration-dependent manner with median inhibition concentration values of 89 pM (95% confidence interval [CI], 44–105 pM) (Ec1-ETA-RRSP) and 361 pM (95% CI, 215–597 pM) (RRSP-DT-Ec1) in MCF7 cells, and 713 pM (95% 546–914 pM) (Ec1-ETA-RRSP) and 310 pM (95% CI, 247–395 pM) (RRSP-DT-Ec1) in HCT116 cells (Figures 2C and 2D). In contrast, the DARPin-RRSP fusion proteins showed no toxicity toward C5120, except for a mild decrease in viability at the highest concentration tested (Figure 2E). Similarly, the controls with the catalytically dead RRSP∗ showed no effect on cell viability (Figures 2C–2E). Notably, the RRSP-DT-Ec1 was significantly more potent than Ec1-ETA-RRSP in MCF7 cells. In contrast, in HCT116 cells it was the other way around, indicating that not only the RRSP, but also the translocation domain contributes to biological efficacy in a cell-type-dependent manner.

### Ras degradation in a tumor-on-a-chip model

**Figure 3:**
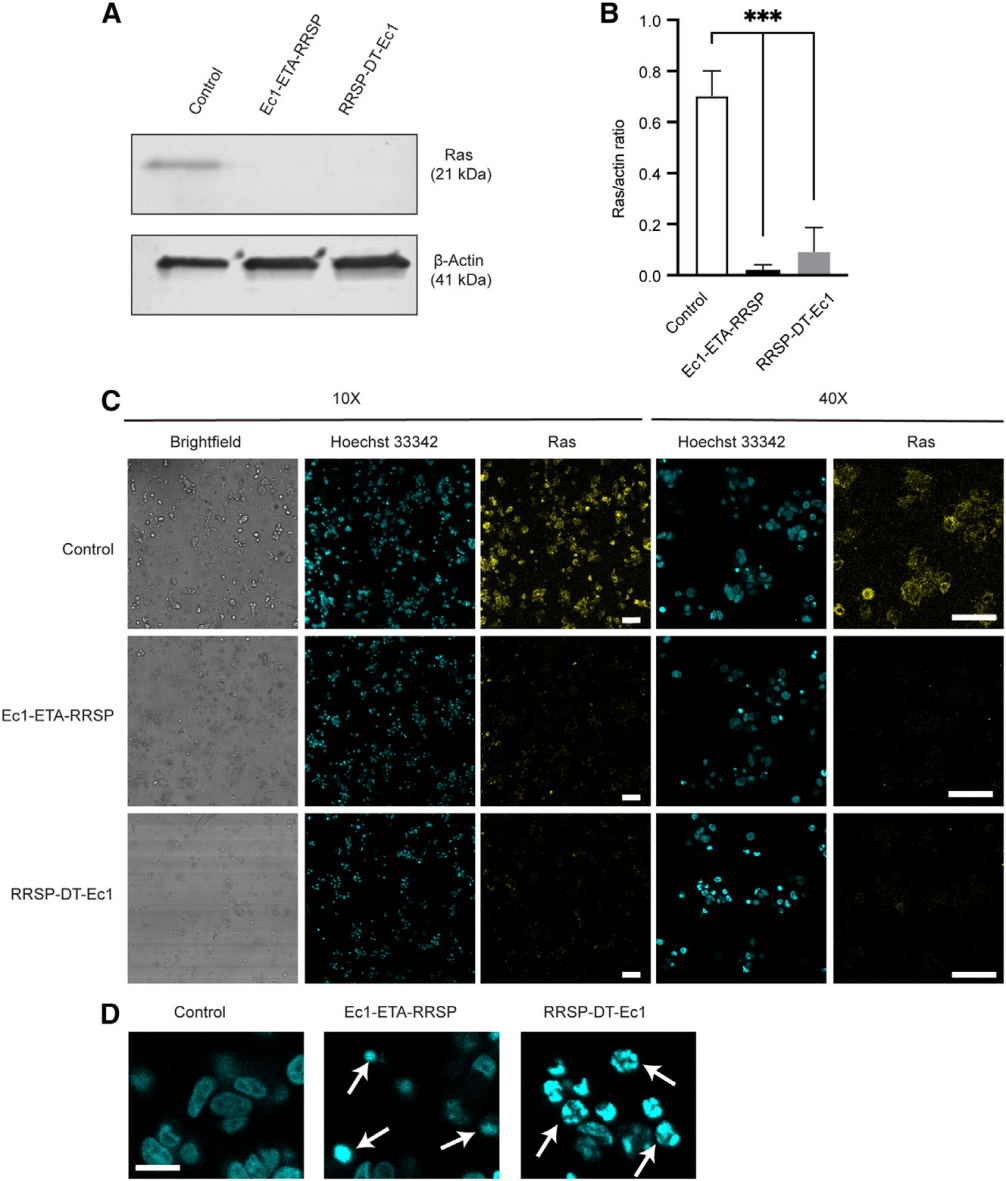
Confirmation of Ras degradation on a microfluidic tumor-on-a-chip (A) Western blot on MCF7 cell lysates after 2 days of treatment with 100 nM Ec1-ETA-RRSP or RRSP-DT-Ec1 on a 3D microfluidic tumor model. The control is untreated; n = 3, representing independent microfluidic devices. β-Actin (42 kDa) was used as a loading control. (B) Western blot quantitative analysis to determine the normalized Ras levels present in the MCF7 lysate. Results are shown as mean values. The error bars reflect the SEM. ∗∗∗p ≤ 0.001, based on a one-way ANOVA (n = 3). The Ras signal was normalized for the intensity of the control β-actin band intensity. (C) Immunofluorescence staining for pan-Ras in MCF7 cells after 2 days of treatment with 100 nM Ec1-ETA-RRSP or RRSP-DT-Ec1 on a 3D microfluidic system. The control is untreated; n = 3, representing independent microfluidic devices. Hoechst 33342 was used as nuclear staining. Scale bars, 100 μm. (D) Zoom-ins from (C). Arrowheads indicate examples of the apoptotic bodies formed. Scale bar, 25 μm.

In MCF7 and HCT116 monolayers, Ec1-ETA-RRSP and RRSP-DT-Ec1 caused complete degradation of Ras as determined by western blotting. Since the 3D microfluidic environment results in a lower diffusion and cell surface receptor accessibility, which is expected to affect efficacy negatively,^9^^,^^17^ we wanted to investigate Ras degradation in a microfluidic tumor model where cells are dispersed in a dense 3D collagen matrix. After 2 days of treatment with 100 nM of the active constructs, degradation of Ras was as complete as it was in the 2D cultures, with essentially no detectable Ras left in the MCF7 cells (Figures 3A and 3B).

To further confirm the effect of the treatment with 100 nM of Ec1-ETA-RRSP or RRSP-DT-Ec1 on the degradation of Ras in the microfluidic tumor-on-a-chip model an immunofluorescence approach was used (Figure 3C). In untreated samples, we observed primarily staining of Ras associated with the plasma membrane, which is in correspondence with the reported localization of Ras.^21^ After treatment with Ec1-ETA-RRSP or RRSP-DT-Ec1, Ras levels were strongly decreased, in agreement with the results from western blotting described above. Furthermore, apoptotic bodies could be observed by the nuclear stain after treatment (Figures 3C and 3D), indicating an efficient apoptosis induction of MCF7 cells on chip. We subsequently assessed the overall viability of MCF7 cells on chip using the resazurin assay (Figure S3). While the viability was decreased, it was not as pronounced as in 2D. Morphological changes were seen after treatment with both toxins, with effects being more pronounced for Ec1-ETA-RRSP.

### Tumor cell-selective Ras degradation in C5120:MCF7 co-cultures on-chip

**Figure 4:**
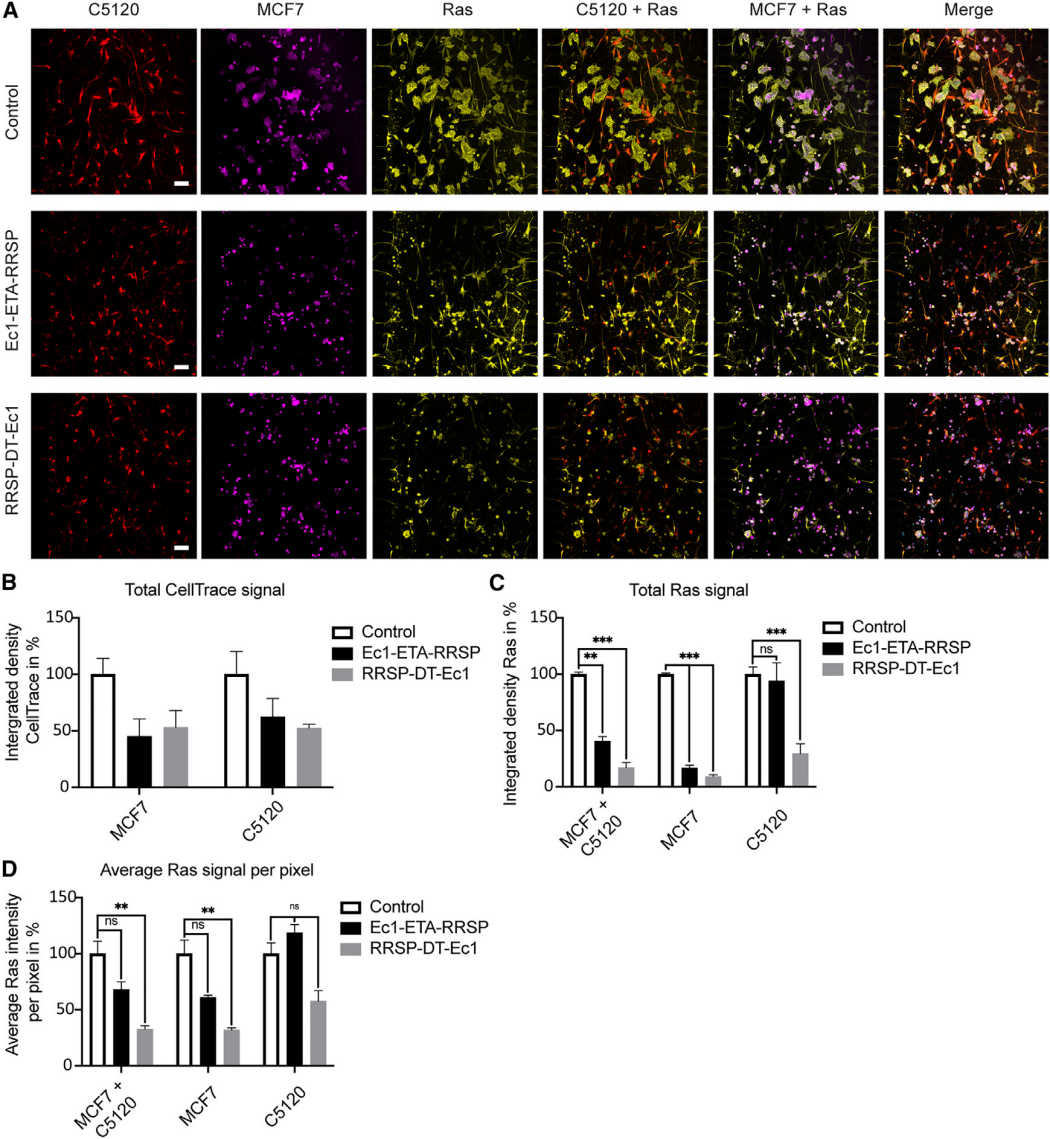
Selective degradation of Ras proteins in tumor cells on co-culture on-chip (A) Immunofluorescence staining for pan-Ras after 2 days of treatment with 100 nM Ec1-ETA-RRSP or RRSP-DT-Ec1 using a C5120:MCF7 co-culture on a 3D microfluidic system. Control was untreated; n = 5, representative images of independent microfluidic devices. Hoechst 33342 was used as nuclear localization marker. Scale bars, 100 μm. (B–D) Quantitative analysis of the microscopy data (10× objective) (n = 3). Results are shown as mean ± SEM. ∗∗p ≤ 0.01, ∗∗∗p ≤ 0.001 based on a two-way ANOVA. (B) Total CellTrace signal measured expressed in percentages compared with control. (C) Total Ras signal present per cell type. (D) Average Ras signal measured per positive pixel (based on CellTrace) to normalize for cell shrinkage.

After proving the ability of Ec1-ETA-RRSP and RRSP-DT-Ec1 to degrade Ras proteins in MCF7 cells in the 3D context, we wanted to test if the constructs could do this tumor cell selectively in co-cultures with normal skin fibroblasts, C5210 cells. The Ras staining of C5120 cells was not affected by treatment with Ec1-ETA-RRSP and only mildly after treatment with RRSP-DT-Ec1 100 nM for 2 days (Figures 4A and S4 and Videos S2, S4, and S6). In MCF7 cells, we observed complete degradation of RAS with either construct throughout the tissue (Figure 4A, Videos S1, S3, and S5). Changes in cell distribution on-chip of MCF7 cells were also observed. Typically, cells form clusters or tumor cell aggregates, but upon treatment with the RRSP fusions, cells were much more isolated and compact, as indicated by both the CellTrace and Ras stains (Figures 4A and S4). Overall, the changes indicate a tumor cell-specific degradation of Ras, leading to a tumor cell-selective induction of apoptosis.

Video S1. Rotating 3D representation of pan-Ras staining in untreated C5120-MCF7 co-culture with MCF7 cells stained Immunofluorescence staining for pan-Ras (yellow) after 2 days of culture on a 3D microfluidic system. MCF7 cells were stained in magenta. The 3D image was reconstructed from Z-stacks acquired by confocal microscopy.

Video S2. Rotating 3D representation of pan-Ras staining in untreated C5120-MCF7 co-culture with C5120 fibroblasts stained Immunofluorescence staining for pan-Ras (yellow) after 2 days of culture on a 3D microfluidic system. C5120 fibroblasts were stained in red. The 3D image was reconstructed from Z-stacks acquired by confocal microscopy.

Video S3. Rotating 3D representation of pan-Ras staining in Ec1-ETA-RRSP-treated C5120-MCF7 co-culture with MCF7 cells stained Immunofluorescence staining for pan- Ras (yellow) after 2 days of treatment with 100 nM of Ec1-ETA-RRSP on a 3D microfluidic system. MCF7 cells were stained in magenta. The 3D image was reconstructed from Z-stacks acquired by confocal microscopy.

Video S4. Rotating 3D representation of pan-Ras staining in Ec1-ETA-RRSP-treated C5120-MCF7 co-culture with C5120 fibroblasts stained Immunofluorescence staining for pan-Ras (yellow) after 2 days of treatment with 100 nM of Ec1-ETA-RRSP on a 3D microfluidic system. C5120 fibroblasts were stained in red. The 3D image was reconstructed from Z-stacks acquired by confocal microscopy.

Video S5. Rotating 3D representation of pan-Ras staining in RRSP-DT-Ec1-treated C5120-MCF7 co-culture with MCF7 cells stained Immunofluorescence staining for pan- Ras (yellow) after 2 days of treatment with 100 nM of RRSP-DT-Ec1 on a 3D microfluidic system. MCF7 cells were stained in magenta. The 3D image was reconstructed from Z-stacks acquired by confocal microscopy.

Video S6. Rotating 3D representation of pan-Ras staining in RRSP-DT-Ec1-treated C5120-MCF7 co-culture with C5120 fibroblasts stained Immunofluorescence staining for pan-Ras (yellow) after 2 days of treatment with 100 nM of RRSP-DT-Ec1 on a 3D microfluidic system. C5120 fibroblasts were stained in red.

Quantitative analysis of the microscopy data showed the reduction of total CellTrace signal, which was most pronounced for MCF7 as expected, yet also evident for C5120 (Figure 4B). Treatment with Ec1-ETA-RRSP or RRSP-DT-Ec1 decreased the total Ras levels in MCF7 cells strongly by 83.2 ± 2.4% (Ec1-ETA-RRSP) and 91.8 ± 1.5% (RRSP-DT-Ec1). The total Ras signal in C5120 cells was not affected by treatment with Ec1-ETA-RRSP but was, remarkably, decreased by 70.5 ± 8.7% after treatment with RRSP-DT-Ec1 (Figure 4C). Because cell shrinkage and growth inhibition would contribute to the decrease in total Ras signal, the intensity of Ras was also quantified per CellTrace-positive pixel, which yielded similar albeit less pronounced effects (Figure 4D).

### Tumor cell-selective toxicity C5120:MCF7 co-culture using propidium iodide staining

**Figure 5:**
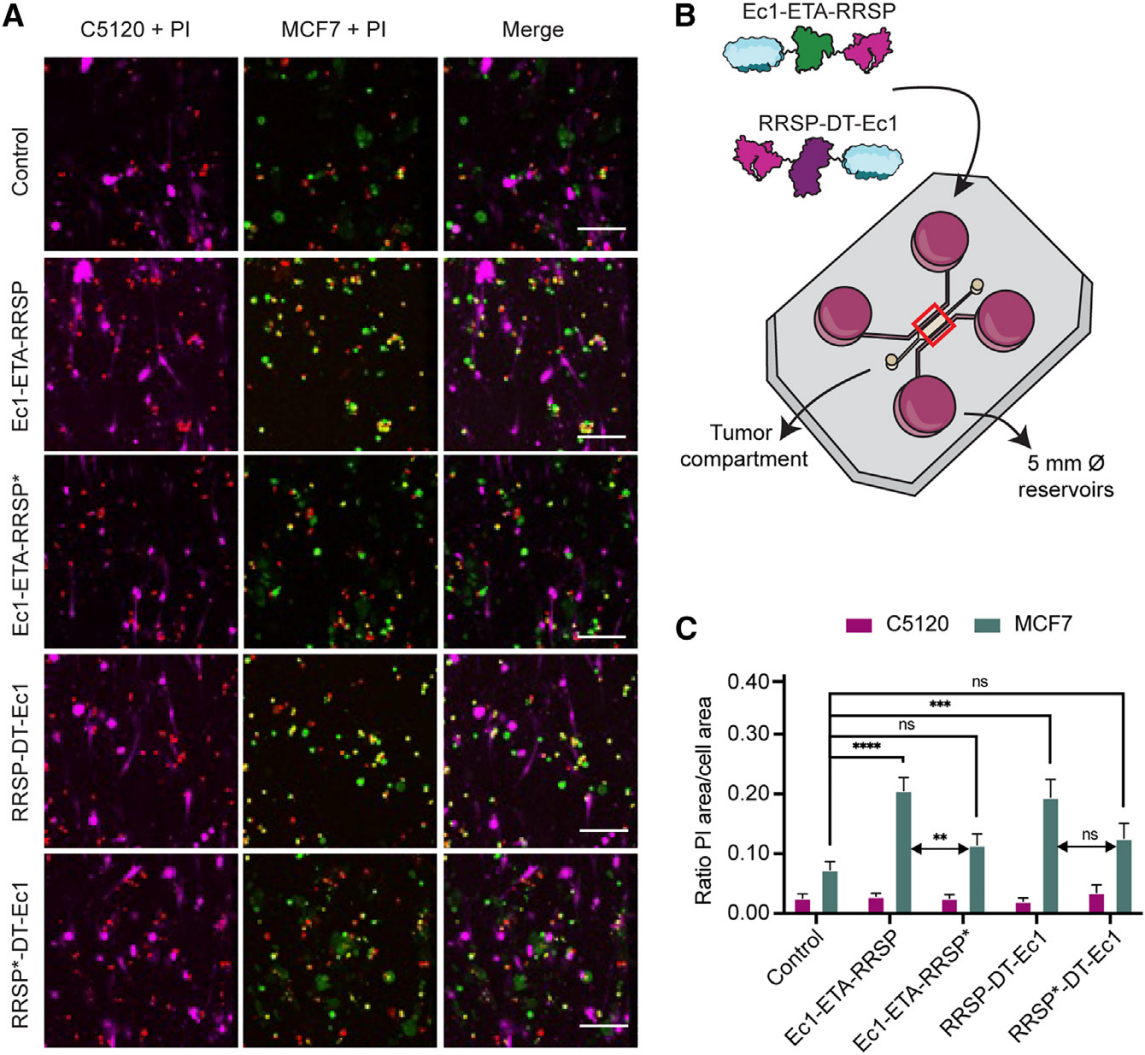
Selective toxicity on chip toward tumor cells with RRSP fusions (A) Microscopy images of the PI staining on C5120:MCF7 co-cultures treated with or without 500 nM Ec1-ETA-RRSP, Ec1-ETA-RRSP∗, RRSP-DT-Ec1, or RRSP∗-DT-Ec1 for a total of 7 days on a 3D tumor-on-a-chip; representative images of n = 6 independent microfluidic devices (n = 5 for RRSP∗-DT-Ec1). RRSP∗ refers to a catalytically dead version of RRSP with the point mutation H451A. Scale bars, 100 μm. The overlapping area between PI (red) and MCF7 (green) results in a yellow pseudocolor. C5120 is depicted in magenta. (B) Schematic depiction of the addition of the proteins to the tumor-on-a-chip and the area imaged for quantification of cell death (red square). (C) Quantitative analysis of the overlapping area between the C5120 signal or MCF7 signal with PI (n = 6) (n = 5 for RRSP∗-DT-Ec1), representing independent microfluidic devices. Results are shown as mean ± SEM. p > 0.05 (ns), ∗∗∗p ≤ 0.001, ∗∗∗∗p ≤ 0.0001 based on a two-way ANOVA.

To test if the constructs could induce tumor-selective toxicity, we tested them on C5120:MCF7 co-cultures in a 3D tumor-on-a-chip system after incubation with 500 nM for 7 days. When looking at the propidium iodide (PI) signal that overlaps with MCF7 cells, which denotes dead tumor cells, there is a clear increase for the active constructs (Figure 5A). No increase in cell death for C5120 fibroblasts was observed. The chips were imaged in the central area encompassing the whole tumor compartment (Figure 5B, red square), and quantification of the overlap between the distinct cell type signal and PI further confirms these findings and shows that only the active constructs significantly increase the amount of tumor cell death, and none of the conditions has an apparent effect on the viability of the C5120 fibroblasts (Figure 5C).

## Discussion

In this study, we successfully demonstrated the selective delivery of a pan-Ras degrader into the cytosol of tumor cells, using two different fusion constructs: RRSP-DT-Ec1 and Ec1-ETA-RRSP. Both constructs effectively degraded Ras proteins in EpCAM-positive cells in both 2D and 3D tumor-on-a-chip systems. However, the ETA-based constructs exhibited enhanced tumor cell selectivity, as observed in the microfluidic tumor model.

Traditionally, targeting Ras proteins has primarily focused on small molecule approaches.^22^ In recent years, there has been an emergence of engineered protein-based methods for Ras targeting. Engineered proteins offer advantages in terms of higher binding specificity and the ability to target large, flat surfaces. Although various binding protein scaffolds have been used to target Ras proteins, their therapeutic potential is limited by their inability to cross the plasma membrane and engage the intracellular target.

To overcome these limitations, delivery platforms and engineering approaches have been developed for Ras-binding proteins to deliver agents that interfere with Ras signaling in the cytosol. These include cell-permeable anti-Ras antibodies,^23^ *Salmonella*-based platforms,^24^ lipid-based nanocarriers,^25^ and the use of the translocation domain of DT for the delivery of the Ras-cleaving enzyme RRSP. While these approaches enable a cytosolic uptake of proteins targeting Ras signaling, they have limited tumor cell specificity and also affect Ras signaling in healthy cells. In our study, we have taken a modular approach that combines the delivery of the Ras-cleaving enzyme RRSP with the ability to redirect its activity toward tumor cells that overexpress a specific marker, EpCAM.

Our system offers modularity not only in the choice of DARPin as a binding protein, but also in the selection of the bacterial toxin-derived translocation domain. We successfully tested our system with translocation domains from both ETA and DT. Importantly, our results showed that the ETA-based construct (Ec1-ETA-RRSP) exhibited higher activity and selectivity compared with the DT-based construct (RRSP-DT-Ec1). The DT-based construct showed decreased Ras signaling in non-targeted fibroblasts, whereas the ETA-based construct did not.

Furthermore, the active RRSP fused to ETA significantly increased cell death compared with the control with inactive RRSP, whereas this increase was not significant for the DT-based constructs. These differences could arise from the cellular stress induced by the DT translocation mechanism^26^ or the effect of binding to EpCAM, which is known to play important roles in various cellular process, such as the regulation of proliferation, differentiation, migration, and cell-cycle processes.^27^^,^^28^^,^^29^ Additionally, the inactive RRSP∗ may still have a residual effect on Ras signaling by blocking Ras signaling through binding to Ras proteins.^7^^,^^18^ In MCF7 and HCT116 cells, a very pronounced effect on cell viability was observed, indicating either a very strong addiction to Ras signaling, or added effects on viability through the ability of RRSP to also degrade Rap1.^30^ A limitation of our study is that our data cannot rule out that other as yet unidentified targets are also affected, which may contribute to the pronounced effects seen on viability.

Immunogenicity is an important factor to consider before applying engineered proteins with bacterial-derived components in humans. Both the translocation domains from ETA and DT, as well as RRSP, are likely to elicit undesirable immune responses. However, efforts have been made to deimmunize these components by removing or suppressing T and B cell epitopes, indicating that immunogenic effects can be mitigated.^31^^,^^32^^,^^33^^,^^34^ Advances in engineering proteins, in combination with immune-modulating drugs, offer potential solutions to address immunogenicity concerns in clinical practice.^35^

In our work, we focused on validating the activity of our engineered constructs in 3D *in vitro* models with human cells, rather than directly moving to animal models from 2D cell culture systems. A key advantage of these models over animal models is that the cellular context is fully human, which is particularly relevant when targeting surface molecules with highly specific binding proteins, as we do here when targeting EpCAM with the DARPin Ec1. Importantly, by using a 3D co-culture microfluidic model that provides a more accurate representation of the human tumor microenvironment, we could identify different specificity profiles of the ETA and DT-based constructs. In general, microfluidic organ-on-chip models enable a more in-depth analysis of specificity, delivery, and molecular activity by having control over crucial factors such as pH, oxygen gradients, cell-matrix interactions, and immune and stromal cell composition.^36^^,^^37^^,^^38^^,^^39^ In previous studies, 3D cell culture models have revealed significant differences in drug sensitivities and gene expression compared with traditional 2D cell culture models because of the influence of matrix mechanics, architecture, and composition on cell adhesion, mechanotransduction, and migration.^40^^,^^41^^,^^42^

Validation of the predictive value of organ-on-a-chip models for *in vivo* outcomes is an ongoing challenge for the field. Recent studies have shown promising results in demonstrating the high predictive value of organ-on-a-chip models for *in vivo* outcomes compared with traditional 2D cell culture models. Ao et al.^43^ recently validated the use of mini-tumor chips using *in vivo* data with the same cells, showing the high predictive value for *in vivo* outcomes of the mini tumors-on-chips, but not the traditional 2D culture. Similarly, Kerns et al.^44^ showed that 2D model fails to recapitulate antibody responses *in vivo*, while organ-on-a-chip successfully predicted *in vivo* outcomes. Our own previous work has also demonstrated the necessity of using 3D models to achieve therapeutic effects, with ≤10,000 times higher effective doses required in 3D models compared with 2D models.^9^ These findings emphasize the importance of employing 3D models in drug development and testing to obtain more accurate and physiologically relevant results.

In conclusion, our study showcases the potential of modular engineered proteins to selectively kill tumor cells by exploiting two layers of selectivity in both 2D and 3D tumor models. The combination of surface molecule targeting (EpCAM) and intracellular target engagement (Ras) offers the potential for higher therapeutic windows and reduced side effects in non-targeted tissues. Additionally, the use of 3D models provides a more realistic representation of the tumor microenvironment and enables in-depth investigations of therapeutic effects. Our results provide a foundation for further development of these agents for clinical applications and serve as a template for the design of similar two-layer targeting agents with improved therapeutic profiles compared with monolayer therapies.

## Materials and methods

### Cell lines and culture conditions

Human breast adenocarcinoma cell lines MCF7 and MDA-MB468 and colorectal carcinoma HCT116 cells (all ATCC, Manassas, VA) were used as cancer cell lines, and C5120 skin fibroblasts^45^ were used as healthy control cells. The engineering of EpCAM-overexpressing FlpIn 293 cells was reported earlier.^16^ All cell culture cultivations were performed in a humidified incubator at 37°C and 5% CO_2_. Cells were cultured in Dulbecco’s Modified Eagle Medium (ThermoFisher Scientific, Waltham, MA), with the exception of C5120 cells, which were cultured in Medium 199 (ThermoFisher Scientific). All media were supplemented with 10% fetal calf serum (FCS) (PAN-Biotech, Aidenbach, Germany) and 1% GlutaMAX (ThermoFisher Scientific).

### Plasmid cloning

MRGS-HIS_6_-Ec1-ETA(252–412)-RRSP-avi-HA-HIS_6_-RDEL was cloned by ligation of a DNA fragment encoding full-length RRSP (Twist Biosciences, South San Francisco, CA) in a previously used pQIq backbone^9^ using SpeI and AgeI restriction sites. As a catalytically inactive negative control, a similar vector encoding RRSP with the mutation H451A (referred to as RRSP∗), with previously confirmed loss of activity,^18^ was generated by PCR and sticky end ligation using the original RRSP construct as a template for the PCR and SpeI and PstI as restriction sites. Similarly, the DT-based constructs were generated by inserting, through EcoRI and NruI sites, a DNA fragment encoding a tobacco etch virus (TEV) cleavage site and RRSP into the pQIq vector MRGS-HIS_6_-TEV-NI_3_C-DT-Ec1,^16^ yielding the TEV-cleavable construct MRGS-HIS_6_-TEV-RRSP-DT(186–378)-Ec1. A catalytically dead negative control as described above was equally generated for the DT-based construct. All sequences were confirmed by Sanger sequencing.

### Protein expression and purification

For protein expression, plasmids were transformed in chemo-competent *E*. *coli* BLR(DE3) cells. Protein expression and subsequent protein purification of HIS_6_-containing constructs were done by IMAC as described before for DARPin-toxin fusion proteins.^9^

### Ras degradation *in vitro* and in 2D monolayer

To study the enzymatic activity of the RRSP fusion proteins, 1 μg pure KRas was incubated with 10 and 100 nM of the engineered proteins in 12 μL for 1 h at 37°C. The samples were then run on gels compatible with stain-free imaging (Bio-Rad, Hercules, CA) using SDS-PAGE and proteins were visualized using a GelDoc EZ imaging system (Bio-Rad). To investigate the Ras-degrading activity over time in 2D monolayers, MCF7 cells were seeded at a density of 150,000 cells per well in 24-well plates and grown overnight. After this, the medium was removed and 100 nM protein in cell culture medium supplemented with penicillin-streptomycin (PS; Merck, Darmstadt, Germany) was added and incubated for 1 h, 2 h, 4 h, and 24 h. After the indicated time points, cells from 24-well plates were detached and centrifuged for 3 min at 300×*g* at room temperature and washed with PBS, and centrifuged again. Protease inhibitor (cOmplete ULTRA tablets, EDTA free; Merck) was added to RIPA buffer (Cell Signaling Technology, Danvers, MA) and the pellet from the PBS wash was resuspended in 50 μL RIPA buffer and placed on ice for 5 min. The lysates were then centrifuged for 10 min at 14,000×*g* at 4°C. The supernatant containing the proteins was collected, snap frozen, and stored at −20°C for further analysis by western blotting as described below. For the concentration-dependent experiments, cells were incubated with indicated concentrations of the DT- and the ETA constructs in culture medium for 4 h, followed by cell lysis with RIPA buffer and processing as described above.

### Detection of Ras levels by western blotting

The loading of an equal amount of protein lysate per well on SDS-PAGE was realized by estimating protein concentrations using a Bradford assay. After SDS-PAGE, blocking of the membrane was done with 1× intercept blocking buffer (Bio-Rad) for 20 min while shaking. The primary antibody diluted 1:2,000 was added after blocking. The antibodies were prepared in a 1:1 [v/v] PBS-T (PBS +0.1% [v/v] Tween 20): intercept blocking buffer solution. After the addition of the primary antibody solution, incubation followed overnight at 4°C while shaking. Pan-Ras monoclonal antibody mouse (ThermoFisher Scientific) and monoclonal anti-beta-actin mouse (Merck) were used as primary antibodies. A secondary antibody solution with the IRDye 800CW Donkey anti-mouse IgG (LI-COR Biosciences, Lincoln, NE) antibody was prepared at a dilution ratio of 1:5,000 in the same buffer, and incubation followed for 45 min at room temperature while protected from light. The PVDF membrane was dried using filter paper and imaged with the Odyssey Clx (LI-COR Biosciences).

### Cell viability assay in 2D monolayer

Resazurin-based viability assays were essentially done as reported before.^9^ Briefly, cells were seeded in flat-bottom 96-well plates at a density of 10,000 cells/well in culture medium supplemented with PS. The next day, the medium was replaced with fresh medium containing the engineered proteins at indicated concentrations. After 3 days, the incubation medium was refreshed, and cells were cultured for 4 additional days. After a total of 7 days, the medium was replaced with a freshly made solution of 0.1 mg/mL resazurin (Merck) in culture medium and incubated for 4 h. Fluorescence of the converted metabolite resazurin to resorufin by viable cells was measured upon excitation at 485 nm and detection at 580–600 nm using the Multimode Plate Reader VICTOR X3 (PerkinElmer, Waltham, MA).

### Fabrication and seeding of tumor-on-a-chip devices

The design of the polydimethylsiloxane-based microfluidic tumor-on-a-chip was previously reported^9^^,^^10^ and consists of one central chamber for cell seeding and two side channels for the addition of medium. The seeding of the cells inside the chips was performed as reported before.^9^

### Detection of Ras levels by immunofluorescence staining

For the experiments with the MCF7 monocultures, the cells were labeled using CellTrace carboxyfluorescein succinimidyl ester (CFSE; ThermoFisher Scientific). In experiments using MCF7 and C5120 co-cultures, cells were labeled using CellTrace Far Red (MCF7) and CellTrace Yellow (C5120) (both ThermoFisher Scientific) essentially as reported before.^9^ The cells were seeded at a 1:1 ratio with a total density of 20 × 10^6^ cells/mL in a collagen-cell suspension. One day after seeding, the medium was replaced with 1 mL of 500 nM of the active RRSP constructs dissolved in culture medium, and incubation for 2 days at 37°C followed. After this time, the incubation medium was removed, and the devices were fixed with 4% paraformaldehyde for 20 min followed by three 5-min washes with PBS. The cells were permeabilized for 10 min using permeabilization solution (0.3% [v/v] Triton X-100 in PBS), followed by 5 min washing with washing solution (4% FCS [v/v] in PBS; freshly prepared). Blocking followed using blocking solution (2% FCS [v/v], 2% BSA [w/v] and 0.1% [v/v] Tween 20 in PBS) for 45 min on the Mimetas rocker mini (Mimetas, Leiden, the Netherlands) set at 0.1 cycles/min. The primary antibody solution of the Pan-Ras monoclonal mouse antibody dissolved 1:200 in blocking solution was added to each reservoir followed by incubation overnight at 4°C on the rocker. After primary antibody incubation, the reservoirs were washed twice for 3 min using the washing solution. After washing, the secondary antibody goat anti-mouse IgG Alexa Fluor 488 (Merck) 1:400 in combination with 1 μg/mL Hoechst 33342 (ThermoFisher Scientific) dissolved in blocking solution was added to each reservoir and incubated for 2 h protected from light on the rocker. After secondary antibody incubation, the reservoirs were washed twice for 3 min using washing solution followed by one wash with PBS. The medium and seeding reservoirs were filled with PBS before confocal microscopy imaging.

### Preparation of lysates from microfluidic experiments for western blotting

The seeding of the microfluidic devices was performed as described above using non-labeled MCF7 cells seeded at a density of 20 × 10^6^ cells/mL in a collagen-cell suspension. One day after seeding, the medium was replaced with 1 mL of 100 nM of the active RRSP constructs dissolved in culture medium, and incubation for 2 days at 37°C followed. The incubation medium was removed and collagenase solution (1 mg/mL; Merck) in culture medium was introduced into the reservoirs. The microfluidic devices were placed in the incubator at 37°C for 10 min to allow collagen degradation. The collagenase-cell suspension was collected, and cells were lysed using RIPA buffer with protease inhibitors. Ras levels were determined by western blotting as described above.

### Cell viability assay in microfluidic device

Microfluidic devices were prepared as described above. MCF7 cells were loaded inside the microfluidic device at a density of 3.0 × 10^6^ cells/mL. Medium was added to all four reservoirs and the chips were placed in the cell culture incubator. After an overnight incubation (day 1), indicated proteins were added at a concentration of 500 nM. Fresh medium with proteins was added at day 3 and day 6, and at day 8 the incubation medium was removed and the resazurin assay was conducted as outlined above.

### Tumor cell-selective toxicity studied by PI staining

The seeding of the microfluidic devices was done as described above. Labeled MCF7 (CellTrace CFSE) and C5120 (CellTrace Violet, ThermoFisher Scientific) cells were seeded 1:1 at a combined density of 10 × 10^6^ cells/mL in a collagen-cell suspension. One day after seeding, the medium was replaced with 1 mL fresh medium containing 500 nM of the active or inactive RRSP constructs, and the cells were incubated for a total of 7 days at 37°C. During the 7 days of incubation, the incubation medium was refreshed after 2 and 5 days of incubation, with fresh constructs added each time. After 7 days, the incubation medium was replaced with culture medium containing 20 μg/mL PI, and cells were further incubated for 1 h at 37°C. The chips were then imaged with confocal microscopy.

### Confocal microscopy

Confocal microscopy was done using the Leica TCS SP8 X white light laser confocal microscope equipped with a temperature-controlled incubator set at 37°C. Sequential imaging was done from bottom to top of the z-compartment with intermediate z-slices of 10 μm. For the cell viability studies using PI, z-stacks of two positions in the microfluidic device were generated. CellTrace Violet was excited at 405 nm (detection, 413–460 nm), Hoechst 3342 at 405 nm (detection, 415–480 nm), CellTrace CFSE at 492 nm (detection, 500–520 nm), PI at 535 nm (detection, 543–620 nm), CellTrace Yellow at 561 nm (detection, 569–611 nm), and CellTrace Far Red at 629 nm (detection, 653–695 nm).

### Image and data analysis

Image analyses were done using ImageJ FIJI*.*^46^ GraphPad Prism 9 was used to plot the figures, extract the median inhibition concentration values and perform statistical analyses. Quantitative analysis of the Western blot data was done by calculating the integrated densities. Normalized Ras/actin ratios were plotted. Statistical analysis on these ratios was done according to a one-way ANOVA. Quantitative analysis of the Ras staining was done by adjusting a macro previously developed in Fiji for evaluating cell death in a tumor-on-a-chip system.^9^ Briefly, the first step was selecting and isolating the z-slice of interest from the z stack. Each distinct fluorophore channel was filtered using a Gaussian blur filter to reduce the background signal. The threshold was determined and the positive signal was converted into regions of interest (ROIs). The overlap between the ROI of Ras and each cell signal ROI was determined and converted into ROIs. The triple overlapping area was determined by creating an ROI of the overlap between the ROIs of Ras, C5120, and MCF7. Using the ROIs, the area, integrated density, and mean signal were measured. The data from the quantitative analysis on the Ras signal were transformed into percentages compared with the control signal. Statistical analysis was done according to a two-way ANOVA. The data obtained from the viability studies using PI was analyzed as described elsewhere.^47^

## Data Availability

The data that support the findings of this study are available from the corresponding author, (W.P.R.V.), upon reasonable request.

## Supplemental information

Document S1. Figures S1–S4

Document S2. Article plus supplemental information
