# Supplementary material for: Potent and selective eradication of tumor cells by an EpCAM-targeted Ras-degrading enzyme
Source: Mol Ther Oncolytics. 2023 Jun 27;30:16–26. doi: 10.1016/j.omto.2023.06.002 (PMC10362089; doi:10.1016/j.omto.2023.06.002)
Supplement: Document S1. Figures S1–S4 [file mmc1.pdf]

OMTO, Volume *30*

Supplemental information

Potent and selective eradication of tumor cells by an EpCAM-targeted Ras-degrading enzyme

Valentina Palacio-Castañeda, Bas van de Crommert, Elke Verploegen, Mike Overeem, Jenny van Oostrum, and Wouter P.R. Verdurmen

Supplemental information

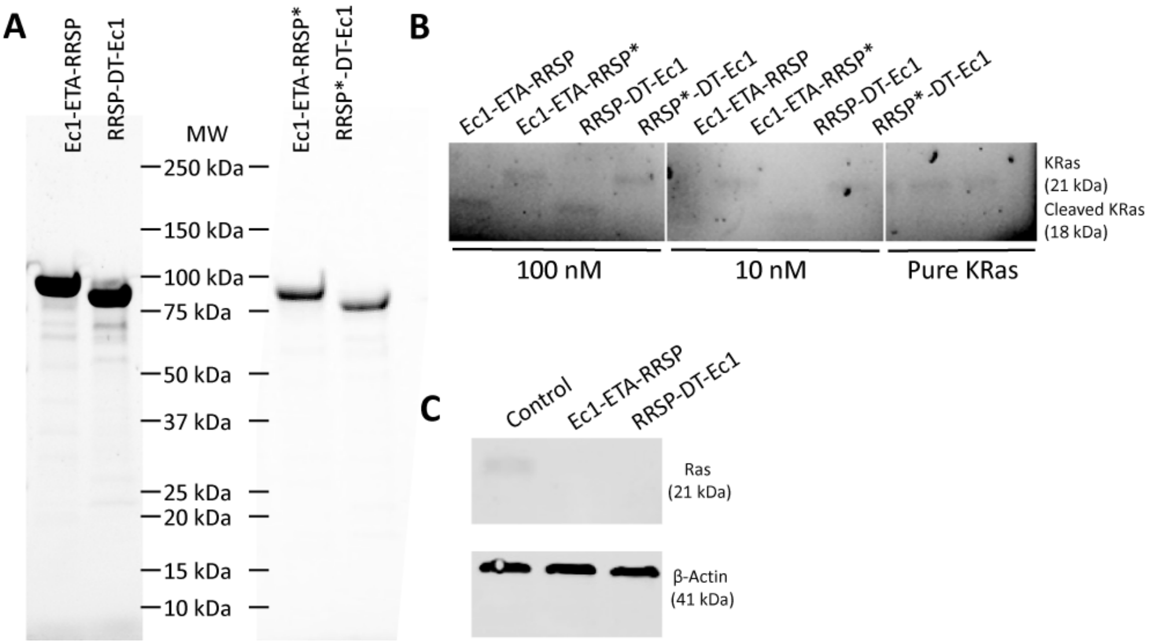

Figure S1. Purity and activity of RRSP fusion proteins. (A) SDS-PAGE of IMAC-purified protein constructs. Bands from Ec1-ETA-RRSP, RRSP-DT-Ec1, and the inactive controls Ec1-ETA-RRSP*, and RRSP*-DT-Ec1 are shown. (**B**) SDS-PAGE of pure KRas incubated with the different RRSP fusion proteins at a concentration of 100 nM or 10 nM. (**C**) Western blot showing degradation of Ras in HCT116 cells after a 24-hour treatment with 100 nM of Ec1-ETA-RRSP or RRSP-DT-Ec1.

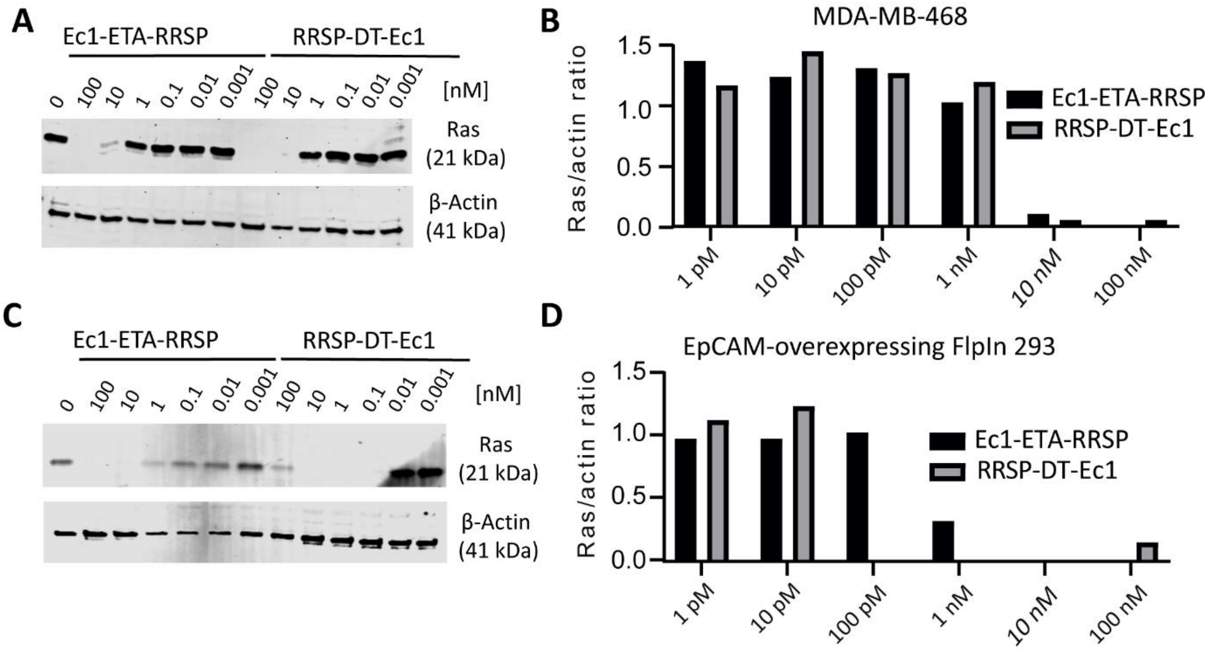

Figure S2. Concentration-dependent Ras degradation in MDA-MB-468 and FlpIn 293 cells. (**A**) Western blot showing degradation of Ras in MDA-MB-468 after a 24-hour treatment with different concentrations of Ec1-ETA-RRSP or RRSP-DT-Ec1. (**B**) Quantification of the Ras/actin ratio from the western blot in (A). (**C**) Experiment as in (**A**), but for EpCAM-overexpressing FlpIn 293 cells. (**D**) Quantification of the Ras/actin ratio from the western blot in (C). Representative western blots are shown, n = 2.

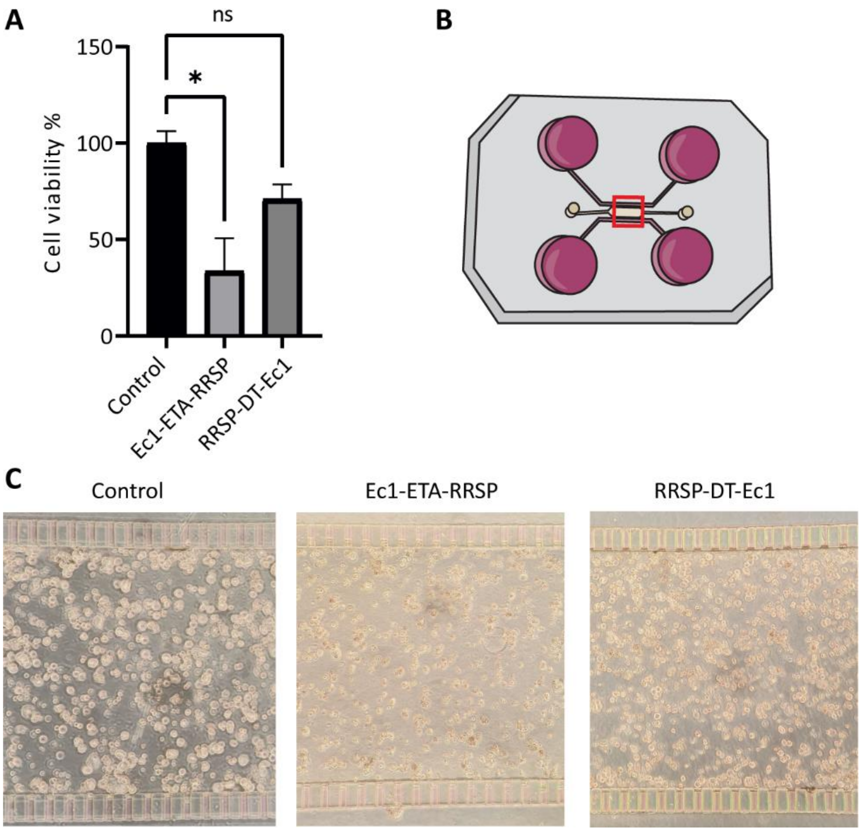

Figure S3. Viability of MCF7 cells in microfluidic devices after treatment with RRSP fusion proteins. (**A**) MCF7 cells were seeded in microfluidic devices and treated for 7 days with 500 nM Ec1-ETA-RRSP or RRSP-DT-Ec1, after which a resazurin was performed to determine cell viability. n = 3, representing independent microfluidic devices. $p \leq 0.05$ (*). (**B**) Schematic drawing of the microfluidic device, with a red square indicating where the pictures in (C) were taken. (**C**) Pictures of the respective conditions were made using Brightfield microscopy.

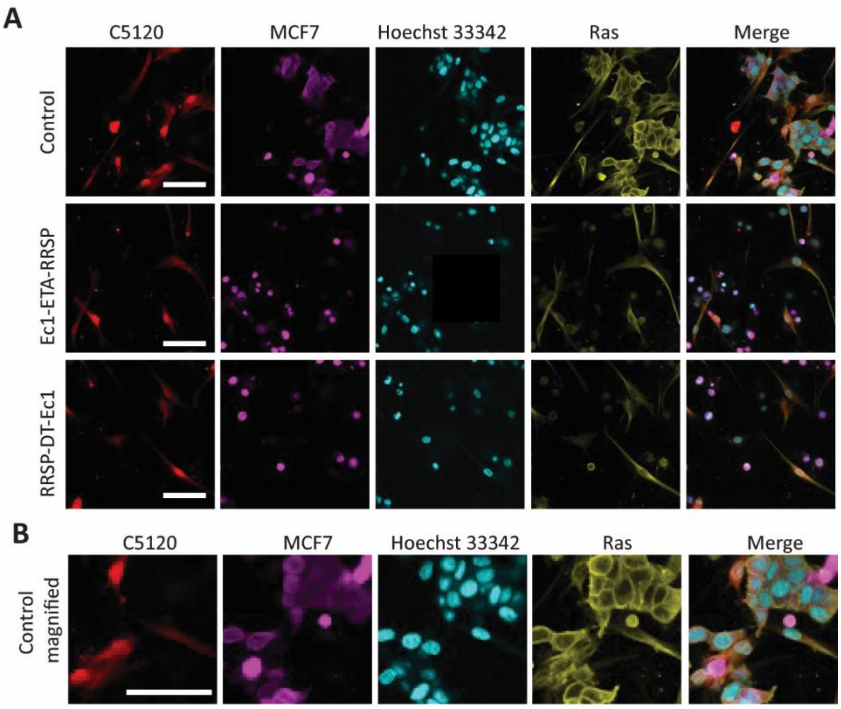

Figure S4. High-magnification of immunofluorescence staining of pan-Ras on a 3D tumor-on-a-chip. (**A**) The C5120:MCF7 co-culture was treated for 2 days with or without 100 nM of Ec1-ETA-RRSP or RRSP-DT-Ec1 on a 3D tumor-on-a-chip system. n=5, representing independent microfluidic devices. Hoechst 33342 was used as nuclear localization marker. Scale bars represent 100 μm. (**B**) Magnified view of the growth of tumor cell aggregates in the control condition in (A).

Video S1. Rotating 3D representation of pan-Ras staining in untreated C5120-MCF7 co-culture with MCF7 cells stained. Immunofluorescence staining for pan-Ras (yellow) after 2 days of culture on a 3D microfluidic system. MCF7 cells were stained in magenta. The 3D image was reconstructed from Z-stacks acquired by confocal microscopy.

Video S2. Rotating 3D representation of pan-Ras staining in untreated C5120-MCF7 co-culture with C5120 fibroblasts stained. Immunofluorescence staining for pan-Ras (yellow) after 2 days of culture on a 3D microfluidic system. C5120 fibroblasts were stained in red. The 3D image was reconstructed from Z-stacks acquired by confocal microscopy.

Video S3. Rotating 3D representation of pan-Ras staining in Ec1-ETA-RRSP-treated C5120-MCF7 co-culture with MCF7 cells stained. Immunofluorescence staining for pan-Ras (yellow) after 2 days of treatment with 100 nM of Ec1-ETA-RRSP on a 3D microfluidic system. MCF7 cells were stained in magenta. The 3D image was reconstructed from Z-stacks acquired by confocal microscopy.

Video S4. Rotating 3D representation of pan-Ras staining in Ec1-ETA-RRSP-treated C5120-MCF7 co-culture with C5120 fibroblasts stained. Immunofluorescence staining for pan-Ras (yellow) after 2 days of treatment with 100 nM of Ec1-ETA-RRSP on a 3D microfluidic system. C5120 fibroblasts were stained in red. The 3D image was reconstructed from Z-stacks acquired by confocal microscopy.

Video S5. Rotating 3D representation of pan-Ras staining in RRSP-DT-Ec1-treated C5120-MCF7 co-culture with MCF7 cells stained. Immunofluorescence staining for pan-Ras (yellow) after 2 days of treatment with 100 nM of RRSP-DT-Ec1 on a 3D microfluidic system. MCF7 cells were stained in magenta. The 3D image was reconstructed from Z-stacks acquired by confocal microscopy.

Video S6. Rotating 3D representation of pan-Ras staining in RRSP-DT-Ec1-treated C5120-MCF7 co-culture with C5120 fibroblasts stained. Immunofluorescence staining for pan-Ras (yellow) after 2 days of treatment with 100 nM of RRSP-DT-Ec1 on a 3D microfluidic system. C5120 fibroblasts were stained in red.
